# Case report: The art of anesthesiology—Approaching a minor procedure in a child with MPI-CDG

**DOI:** 10.3389/fphar.2022.1038090

**Published:** 2022-12-15

**Authors:** En-Che Chang, Yu-Hsuan Chang, Yu-Shiun Tsai, Yi-Li Hung, Min-Jia Li, Chih-Shung Wong

**Affiliations:** ^1^ School of Medicine, Fu-Jen Catholic University, New Taipei, Taiwan; ^2^ Department of Pediatrics, Cathay General Hospital, Taipei, Taiwan; ^3^ Department of Anesthesiology, Cathay General Hospital, Taipei, Taiwan; ^4^ Graduate Institute of Medical Science, National Defense Medical, Taipei, Taiwan

**Keywords:** CDG, ketamine, sevoflurane, hypoalbuminemia, neuromuscular blocking agents, hypotonia, intravenous flushing

## Abstract

**Background:** Protein glycosylation plays an important role in post-translational modification, which defines a broad spectrum of protein functions. Accordingly, infants with a congenital disorder of glycosylation (CDG) can have N-glycosylation, O-glycosylation, or combined N- and O-glycosylation defects, resulting in similar but different multisystem involvement. CDGs can present notable gastrointestinal and neurologic symptoms. Both protein-losing enteropathy and hypotonia affect the decision of using anesthetics. We reported a case of MPI-CDG with protein-losing enteropathy and muscular hypotonia that underwent different anesthesia approach strategies of vascular access. Here, we highlight why intubation with sevoflurane anesthesia and sparing use of muscle relaxants is the optimal strategy for such a condition.

**Case presentation:** A 25-month-old girl, weighing 6.6 kg and 64 cm tall, suffered chronic diarrhea, hypoalbuminemia, and hypotonia since birth. Protein-losing enteropathy due to MPI-CDG was documented by whole-exome sequencing. She underwent three sedated surgical procedures in our hospital. The sedation was administered twice by pediatricians with oral chloral hydrate, intravenous midazolam, and ketamine, to which the patient showed moderate to late recovery from sedation and irritability the following night. The most recent one was administered by an anesthesiologist, where endotracheal intubation was performed with sevoflurane as the main anesthetic. The patient regained consciousness immediately after the operation. She had no complications after all three sedation/anesthesia interventions and was discharged 7 days later, uneventful after the third general anesthesia procedure.

**Conclusion:** We performed safe anesthetic management in a 25-month-old girl with MPI-CDG using sevoflurane under controlled ventilation. She awoke immediately after the procedure. Due to the disease entity, we suggested bypassing the intravenous route to avoid excess volume for drug administration and that muscle relaxant may not be necessary for endotracheal intubation and patient immobilization when performing procedures under general anesthesia in CDG patients.

## 1 Introduction

The provision of safe anesthesia for pediatric patients depends on a clear understanding of the physiologic, pharmacologic, and psychological differences between children and adults (Miller et al., 2009). Practitioners should evaluate several critical pre-operational conditions when planning for pediatric anesthesia. These medical conditions include but are not limited to a patient’s developmental status, the aim of the surgery, preoperative preparation, pharmacology of the candidate drugs, and the possible side effects after administration (Miller et al., 2009). Every step matters; the more information collected, the better the plan can be made. This is particularly important when it comes to dealing with rare diseases. Infants with CDGs present varying levels of involvement of the central nervous system (most often hypotonia and ataxia), dysmorphology, gastrointestinal symptoms including protein-losing enteropathy, and other signs (Kliegman et al., 2016). Some symptoms affect the choice of anesthesia management, while facial dysmorphism/preterm birth raises worries of airway insecurity; both protein-losing enteropathy and hypotonia can affect the decisions made during anesthesia management. To date, the literature on this subject lacks reports on anesthesia management in children with CDGs. To date, only three reports have been published, each regarding CDG types, namely, PMM2-CDG, ALG6-CDG, and STT3B-CDG (Sakai et al., 2017) (Meaudre et al., 2005) (Lehavi et al., 2011). Sakai et al. (2017) suggested the use of neuromuscular monitoring when using rocuronium on CDG patients with hepatic dysfunction and hypotonia. Meaudre et al. (2005) highlighted the complexity of coagulopathy in CDG patients and the perioperative assessment of clotting factors, as part of which practitioners use fresh frozen plasma or a prothrombin complex concentrate to lower hemorrhagic risk during surgery (Altassan et al., 2019). Lehavi et al. (2011) described a nitrous oxide–remifentanil-based anesthesia on a 6-year-old boy (16.2 kg), taking into account concerns about the CDG patient’s hemodynamic status and hepatic function. At present, there are no specific guidelines for managing anesthesia in CDG patients, and more data is required. In response, the present study reports on the anesthesia practices used for a 25-month-old girl suffering from MPI-CDG with protein-losing enteropathy and muscular hypotonia. In our case, we present three approaches to anesthesia management during vascular access for regular albumin infusion, performed by different specialists. We also discuss the particular concerns of the approaches and propose an overall strategy.

## 2 Case presentation

**FIGURE 1:**
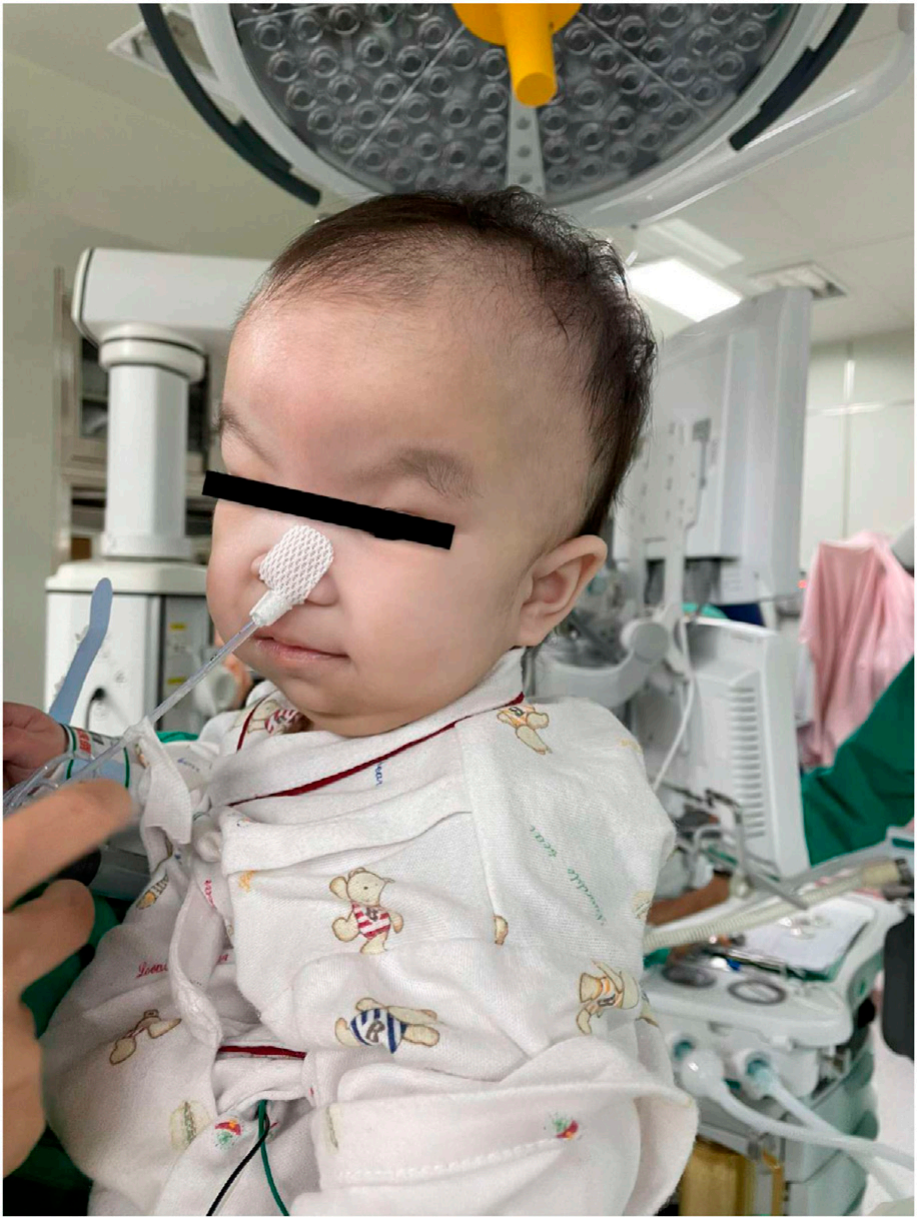
Photo of the patient before the port-a-cath exchange. Her dysmorphic features were wide prominent forehead, flat nose, large anterior fontanelle, web neck, and skeletal dysplasia.

The patient was a 25-month-old girl, who weighed 6.6 kg ( < 0.1 percentile) and was 64 cm ( < 0.1 percentile) tall. She was diagnosed with MPI-CDG with protein-losing enteropathy, hypoalbuminemia, hypogammaglobulinemia (IgG = 118.0 mg/dl), muscular hypotonia, and some dysmorphic features, including a wide prominent forehead, flat nose, large anterior fontanelle, web neck, and skeletal dysplasia (Figure 1). Her liver transaminase levels were within normal limits (AST = 17 IU/L; ALT = 14 IU/L). She underwent three sedated vascular access surgeries for regular albumin infusion in our hospital. The sedation was administered twice by pediatricians to place a peripherally inserted central catheter (PICC), and the most recent one was administered by anesthesiologists for a port-a-cath exchange. The pediatricians gave oral chloral hydrate, intravenous midazolam, and ketamine while monitoring vital signs including body temperature (BT), blood pressure (BP), heart rate (HR), arterial oxyhemoglobin saturation (SaO_2_), and respiratory rate (RR). On the other hand, the anesthesiologist performed endotracheal intubation and gave sevoflurane inhalation with oxygen while monitoring similar vital signs including end-tidal carbon dioxide (ETCO_2_) for airway and respiratory function monitoring.

The first sedation was administered by pediatricians in the pediatric intensive care unit (PICU) for the purpose of placing a PICC. Routine non-invasive monitoring was established, including BT, BP, HR, SaO_2_, and RR. Before anesthesia, her vital signs were stable: BT: 36.4°C, BP: 95/74 mmHg, HR: 114/min, SaO_2_: 100%, and RR: 34/min. The sedation was induced orally using a 3.5 ml 10% chloral hydrate solution (0.5 cc⋅ *kg* ^−1^), intravenous 0.7 mg midazolam (0.1 mg ⋅ *kg* ^−1^), and continued on 6 mg ketamine (0.9 mg ⋅ *kg* ^−1^) seven times, with a total dosage of 42 mg within 169 min. The surgery lasted 215 min; however, we failed to insert the PICC. During the procedure, the addition of ketamine was adjusted according to restlessness to ensure that the patient did not wake up. The patient’s vital signs remained stable, with no bradycardia, hypotension, apnea, or arterial desaturation, during the whole procedure. After awakening from the procedure, there were no other symptoms or discomfort for the patient, except for appearing irritable the following night.

The second sedation was also administered by pediatricians in the PICU to place a PICC. The procedure was performed by a plastic surgeon, who asked for a completely stable patient during the operation. Routine monitoring was established, including BT, BP, HR, SaO_2_, and RR. Before the anesthesia, her vital signs were stable: BT: 36.2°C, BP: 91/57 mmHg, HR: 120/min, SaO_2_: 99%, and RR: 30/min. Prior to the procedure, she was induced using 3.4 ml 10% chloral hydrate solution (0.5 cc⋅ *kg* ^−1^), 0.7 mg midazolam (0.1 mg ⋅ *kg* ^−1^), and continued on 6 mg ketamine (0.9 mg ⋅ *kg* ^−1^) five times, with a total dosage of 30 mg within 147 min. The surgery lasted 90 min, anesthesia time was 170 min, and a heart rate over 150/min was used as a sign of awakening and for additional drug dosage supplements. The patient remained stable, and a PICC was successfully placed. During the operation, her vital signs were also stable, with no bradycardia, hypotension, apnea, or arterial desaturation. There were no other complications or discomfort from the patient except for appearing irritable the night after the administration of anesthesia.

The latest anesthesia was administered by anesthesiologists for a port-a-cath exchange in the operating room. At the pre-anesthesia assessment, her anesthesia status was graded as American Society of Anesthesiologists (ASA) Class III for CDG disease entity and possible difficulty in airway establishment. The laboratory examination showed hypoalbuminemia and anemia, and her chest X-ray showed increased non-specific infiltrate in the bilateral lower lungs. Electrocardiography showed normal sinus rhythm. Before the anesthesia, her vital signs were stable with BT: 36.2°C, BP: 89/47 mmHg, HR: 119/min, SaO_2_: 99%, and RR: 50/min. In order to minimize complications from endotracheal intubation, GlideScope® was used, and the patient was brought to a sniffing position with neck protection. After successful airway establishment, sevoflurane was used as the main anesthesia. After evaluating the patient’s muscle tone, muscle relaxants were not administered. A total of 0.1 mg atropine was given to maintain the heart rate (150–160/min) for an adequate cardiac output. The surgery lasted 60 min, and the anesthesia time lasted 120 min. The patient’s vital signs remained stable and under secured ventilation control, with no bradycardia, hypotension, apnea, or arterial desaturation. After completion of the operation, the patient regained consciousness and was sent back to the PICU, where the ET tube was removed and a nasal cannula was placed for 4 h. No complication or discomfort was observed, and the patient was discharged uneventfully 7 days later.

## 3 Discussion

### 3.1 Overview

Three anesthetic interventions were performed in our reported MPI-CDG case. When facing rare diseases, the common obstacles for healthcare professionals include diagnostic delays and lack of information and treatment options. A comprehensive evaluation is particularly critical in situations like this. In this case, the anesthesiologists routinely and thoroughly evaluated the patient’s condition, especially included assessing the patient’s airway condition. While each of the patients’ experiences under anesthetic were fine, it is important to remember that the aim of using anesthesia is to ease the patient for an unbothered surgery.

### 3.2 Airway management

When managing children under anesthesia, it is important to constantly maintain a secured airway with satisfactory ventilation and oxygenation. A failed airway can cause hypoxia, potentially leading to brain damage and death within minutes (Cook and MacDougall-Davis, 2012). It is currently reported that more than half of critical perioperative events in children are respiratory complications (Habre et al., 2017). Any improvement in preparation (mainly preoxygenation and patient positioning), intubation techniques, and removal of airway devices can minimize perioperative complications. Additionally, capnography monitoring for critical information on ventilation, perfusion, and metabolism is a standard tool that ensures the establishment of a secured endotracheal tube (Soto et al., 2004). In cases in which difficulties with the airway might be expected, it is recommended that intravenous access be prepared beforehand for instant management of potential emergencies, e.g., laryngospasm and bradycardia, where the latter is usually prevented by administering 0.02 mg/kg atropine (Karsli, 2015). In addition, experts recently recommended the use of video laryngoscopy as the first option for patients in which intubation is anticipated to be difficult (Dadure et al., 2019).

The concerns mentioned above were addressed in the third operation. In this case, we anticipated difficult airway management because of dysmorphism (low-set ears, wide eye distance, and some retrognathia) (Roth et al., 2021) and a narrow airway evidenced by X-rays. Preoxygenation and a neutral airway position were established, atropine 0.1 mg was administered through pre-established PICC, and video laryngoscopy (GlideScope^®^) was used for tracheal intubation. The successful airway establishment and monitoring of ETCO_2_ not only secured the patient’s status for surgery but also suited the chosen anesthesia route, which is discussed in the next section.

### 3.3 Anesthetic administration

The induction of general anesthesia for children occurs either by inhalation or intravenously (IV). While inhalation induction is the most common technique in young children, there are several conditions for which IV induction is preferred. Whatever the route, it is always necessary to evaluate the children’s history and laboratory findings before planning the anesthetic to be used. We discussed the concerns in three dimensions: patients’ laboratory status, ketamine experience and pharmacokinetics, and IV route drawbacks.

First of all, Saad et al. (2020) strongly highlighted the importance of preoperative malnutrition screening and management for the risk of anesthetic overdose. In our case, the girl has hypoalbuminemia with underlying protein-losing enteropathy. Although supported by intravascular nutrient support, her preoperative albumin levels were all low. The high prevalence of hepatic dysfunction in CDG patients is also noticeable (da Silva et al., 2017) (Schollen et al., 2004), with her transaminases within normal limits but prothrombin time shortened. These laboratory findings affect the pharmacokinetics of anesthetics, in this case, ketamine.

In children, ketamine plays an anesthetic role in short-term procedures. Well-known for its psychodysleptic effects, ketamine is a rapid-acting N-methyl-D-aspartic acid (NMDA) receptor non-competitive antagonist (Kurdi et al., 2014). It also interacts with opioid receptors, monoamine, cholinergic, purinergic, and adrenoreceptor systems, providing both positive and negative modulation in sedation and analgesia (Nowacka and Borczyk, 2019). The benefits include preserving children’s cardio-respiratory stability by enhancing or maintaining a normal skeletal muscle tone (Rosenbaum et al., 2021). However, this does not ensure a secured airway and there may be transient minimal respiratory depression if ketamine is administered too rapidly or in too high a dose. Therefore, pharmacokinetics should always be evaluated on a case-by-case basis. According to the literature (Trevor et al., 2019) (Miller et al., 2009), ketamine onset occurs rapidly due to high lipid solubility and ceases its effect by redistribution to inactive sites at a half-life of 11–16 min. Two aspects of ketamine properties were the foci in our case. For one, it is metabolized in the liver through N-demethylation by the cytochrome CYP3A4 (Dinis-Oliveira, 2017). Second, ketamine is the only intravenous anesthetic that has low protein binding (approximately 12%). In our experience, pediatricians empirically chose IV ketamine with midazolam adjuvant for two sedation episodes. Propofol was not used for her age, under 3, according to the Food and Drug Administration (FDA) of the United States. Although both single and accumulative ketamine dosage were within normal limits (induction 0.5–2 mg/kg, lethal dose 600 mg/kg (Orhurhu1 et al., 2021)), the supplement interval (mean 25 min) indicated a mildly prolonged sedation. We tried to explain this outcome despite the lack of literature on the two foci, liver metabolism and protein binding mentioned above. Given that the girl showed normal transaminase levels, we considered there to be a low risk of hepatic-derived complications in the anesthesia outcome. On the other hand, hypoalbuminemia has a great effect on high protein-binding agents; since ketamine is one of the low protein-binding anesthetics, ketamine pharmacokinetics are rarely studied in hypoalbuminemia. However, as hypoalbuminemia more or less reduces protein-binding and increases the free active fraction of drugs (Pino, 2019), we considered this as the cause of her two prolonged ketamine sedations. Last, ketamine-induced dissociation is a major side effect causing concern in pediatric anesthesia. Although midazolam was used as an adjuvant to reduce ketamine induction dosage, the child showed irritability after both interventions. Therefore, the decision to use ketamine should be made carefully, especially when the child shows abnormal susceptibility.

Residual drugs can remain in the dead space of intravenous lines, which is especially crucial when giving small-volume infusions ( < 250 ml), according to the National Infusion and Vascular Access Society (NIVAS, 2021). However, there are debates about whether it is necessary to flush, due to there being a lack of evidence. The current guidance (NIVAS, 2019) provides three options, including discarding the infusion set, flushing manually with 50 ml sodium chloride (0.9%), and flushing with a closed system using an additional fixed needle free connector at the top of the given set. Heparin is also used to prevent intraluminal clot formation and/or catheter colonization (Goossens, 2015). Recently, experts (Rout et al., 2020) (Cousins, 2018) have raised concerns about underdosing, claiming that most healthcare organizations chose option one rather than flushing to cut costs. This is supported by Harding et al. (2020) who found out that up to 35% of medication may not be administered due to residual volume, with the greatest percentage associated with 50-ml solutions. In our case, the girl received seven and five ketamine IV bolus at 0.12 ml per dose (Ketalar^®^500mg/10 ml, 6 mg per dose) in her first two operations, which fits the definition of small-volume infusion. According to NIVAS, in this case, the best practice that minimizes medication loss would be to flush the cannula before and after the drug administration, which in our general practice will be twice the catheter dead space volume. When planning her third anesthesia, we considered not using the intravenous route for two reasons. For one, she had undergone numerous intravenous procedures since her birth, which brought concerns about her peripheral vessel patency. Second, given the dead space (2 ml) in her PICC and the small volume of her dosage (0.12 ml), flushing for complete administration was necessary. Unfortunately, this may have brought about fluid overload when repeated doses were needed in such a small baby (6.6 kg) with hypoalbuminemia. According to Holliday and Segar (1957), the fluid rate for full maintenance of a 6.6-kg child is approximately 26.4 ml/h. If we applied the practice mentioned previously, we would have flushed an estimated 30 ml, an amount beyond full maintenance, which meant that the calculated fluid overload percentage, using the definition developed by Goldstein et al. (2001) or the Goldstein method, would have increased by 1.8%, with every 1% increase in the odds ratio by 1.04 (Selewski et al., 2011). On the other hand, the induction of inhalation agents depends primarily on gas flow or controlled ventilation in children. Taking advantage of the secured intubation we established beforehand and preserving her heart rate with atropine, we chose sevoflurane as the anesthetic for our third operation, in addition to the common benefits of the drug. Finally, the inhalation route also strongly supports the maintenance of sedation for the entire duration of the operation and avoiding intravenous flushing with its realted concerns.

### 3.4 Hypotonia and muscle relaxants

Muscle relaxants, or neuromuscular blocking agents (NMBAs), are commonly used in anesthesia under the indication to facilitate intubation and surgery condition. However, the use of NMBAs should be determined individually (Gueret et al., 2004). NMBAs can have an unexpectedly prolonged effect in patients with hypotonia, and the susceptibility of patients with CDG to non-depolarizing NMBAs remains unclear (Sakai et al., 2017). Although it is not well understood, to the best of our knowledge, trans-synaptic signaling is reduced in CDGs (Frappaolo et al., 2018), the expression of postsynaptic nicotinic acetylcholine receptors with normal function is also reduced (Freeze et al., 2014). This supports our assumption in treating CDG as myasthenia gravis when it comes to NMBAs. For most surgical procedures, administration of NMBAs is not necessary for patients with myasthenia. Adequate relaxation is often reached using inhalation agents alone (Nitahara et al., 2007) (Rocca et al., 2003), in our case, sevoflurane. Even if NMBAs are needed, due to susceptibility concerns (Eisenkraft et al., 1988), it has been suggested that non-depolarizing NMBAs should be used, namely rocuronium or vecuronium with a reversal with sugammadex (Tsukada et al., 2021) (de Boer et al., 2014). However, sugammadex has not received FDA approval for use in children, and to date, there are limited data regarding its administration to pediatric patients (Tobias, 2017). Therefore, due to the patient’s hypotonia and adequate relaxation after administering sevoflurane, additional muscle relaxants were not required to achieve the degree of immobilization required for the surgeon to proceed with the port-a-cath replacement.

### 3.5 Summary and strategy

**FIGURE 2:**
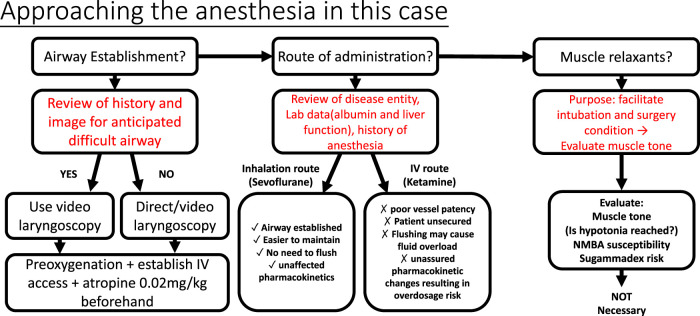
Approach to the anesthesia in the case. Secured airway prevents respiratory complications and brain hypoxia in children’s anesthesia. After the review of X-ray and evaluating child appearance, video larnygoscopy was chosen for expected difficult airway intubation. Decision of the route of administration depends on the pros and cons. Hypoalbuminemia increases free active fraction of protein-bound drugs; at the same time, elevated liver transaminases/liver pathology may also interfere with drug metabolism, in this case, ketamine. On the other hand, anesthetic sevoflurane was eliminated directly *via* lung exhalation, bypassing the liver metabolism. Therefore, the inhalation route outweighed the IV route with its convenience and safety. Muscle relaxants act to facilitate endotracheal intubation and operation; in this case, with hypotonia and the muscle relaxation effect of sevoflurane, NMBAs were not necessary. Sugammadex is a good choice for immediately reversing non-depolarizing muscle relaxants (rocuronium or vecuronium). However, it has not been approved by the USFDA for use in children under two years of age.

The pharmacological characteristics of good sedation and analgesia include ease of application, rapid action, short duration of action, and lack of significant adverse reactions (Norambuena et al., 2013). An experienced and professional practitioner can be a valuable asset when approaching unclear situations (Schulz et al., 2013). The cases presented here raise three crucial concerns of anesthesia, including airway management, choice of drug, and the decision to use a muscle relaxant. Although there is a lack of precise guidelines for anesthesia in CDGs, decisions can still be made by a comprehensive strategy (Figure 2) including evaluating the patient, understanding the underlying mechanisms, and weighing the medical risks and benefits, all to achieve the final purpose—to assure the surgeon and secure the patient.

## 4 Conclusion

This study reports rare clinical experiences in MPI-CDG children’s anesthesia management. Comparing the three anesthetic experiences, we have reviewed the decisions made by two specialists (Table 1). Based on our review of the literature and discussion of recent studies, we suggest that when anesthesia is needed for CDG patients practitioners use inhaled anesthetics instead of the intravenous route and consider not using NMBAs if patients show hypotonia. These findings will help in administering an anesthetic to CDG children in the future and we encourage further research on this subject.

**TABLE 1:** Comparison of the three anesthetic practices. Notice the durations of both ketamine-induced anesthesia and hypoalbuminemia status, which raise overdose and fluid overload concerns.

	Anesthesia by pediatrics for CVC (9/22)	Anesthesia by pediatrics for PICC (10/08)	Anesthesia by anesthesiologist for port-a-cath (10/11)
Duration of anesthesia	215 min (14:50–18:25) BW:6577 g	170 min (15:30–18:20) BW:6692 g	120 min (8:10–10:10) BW:6600 g
Anesthetics and dose			
General	Chloral hydrate soln. 10% dose 3.5 ml at 0 min, midazolam 15 mg/3 ml dose 0.7 mg at 20 min, ketamine 500 mg/10 ml dose 6 mg at 36 min, 100 min, 128 min, 154 min, 178 min, 192 min, and 205 min, respectively (seven times totally)	Chloral hydrate soln. 10% dose 3.4 ml at 0 min, midazolam 15 mg/3 ml dose 0.7 mg at 45 min, ketamine 500 mg/10 ml dose 6 mg at 55 min, 57 min, 89 min, 134 min, and 202 min, respectively (five times totally)	Sevoflurane 4L/min from 0 min to 5 min (5 min, Induction), sevoflurane 2L/min from 5 min to 90 min (85 min, maintain) O_2_ 2L/min from 0 min to 120 min
Local	Lidocaine HCl 2% dose 20 ml at 15 min	None	None
Muscle relaxants	None	None	None
Others	None	None	Atropine 0.1 mg
Vital sign			
Before anesthesia	BT: 36.4, HR: 114, SaO_2_: 100, and RR: 34	BT: 36.2, HR: 120, SaO_2_: 99, and RR: 30	BT: 36, HR: 119, SaO_2_: 99, and RR: 50
During anesthesia	Vital signs stable, BT: 36.7, HR: 124 ∼128, SaO2: 98 ∼100%, and RR: 43 ∼53	Vital signs stable, BT: 36.2 ∼37.0, HR: 120 ∼135, SaO2: 99 ∼100%, and RR: 30 ∼54	Vital sign stable, BT: 36.5 ∼37.0, HR: 145 ∼150 ET intubation with control ventilation: ETCO_2_: 41 ∼52, SaO_2_: 100%
After anesthesia	BP: 84/54 mmHg, BT: 36.7 ∼37.9, HR: 154, SaO_2_: 100, RR: 39, and emotional irritation for one night	HR: 150, SaO_2_: 100%, RR: 42, and emotional irritation for one night	One vomiting episode after arriving from the PICU while on an ET tube, fair spirit after removal of the ET tube intermittently asleep/awake, nasal cannula flow rate: 0.5L/min, BT: 36.9, HR: 146, BP: 81/66, SaO_2_: 100, and RR: 29 ∼51
Lab	Albumin: 2.3 g/dl (L, 9/20) and albumin: 2.4 g/dl (L, 9/27)	Albumin: 1.4 g/dl (L, 10/04) and albumin: 1.8 g/dl (L, 10/09)	Albumin: 1.8 g/dl (L, 10/09) and albumin: 3.2 g/dl (L, 10/09)
Comments	Not suggested due to 1) overdose and flushing concerns. 2) Ketamine: pharmacokinetics and side effect. 3) IV drug supplement when the child shows signs of awakening, interrupting the surgery. 4) Not intubated		Suggested: 1) intubated with secured airway. 2) Secured anesthesia with an inhalation route

## Data Availability

The original contributions presented in the study are included in the article/Supplementary Material; further inquiries can be directed to the corresponding authors.
